# 694 Hand Modeling: A Novel Approach to Orthosis Fabrication of the Pediatric Burnt Hand

**DOI:** 10.1093/jbcr/iraf019.323

**Published:** 2025-04-01

**Authors:** Christopher Whitehead, Christina Mooneyham

**Affiliations:** Shriners Children’s – Galveston; Shriners Children’s - Texas

## Abstract

**Introduction:**

The fabrication of hand orthoses for the pediatric population is challenging due to the intricacies of small hands as well as the limited comprehension of the patients. It is further complicated by poor skin integrity, pain, anxiety, and hypersensitivity to temperature making it difficult to achieve an orthosis that efficiently complements the contours of the hand. These factors can lead to a poor fitting orthosis making the patient at high risk for skin breakdown, loss of range of motion, and discomfort. Our purpose was to explore using a 3D printed model of a patient’s hand as a mold to create a highly customized orthosis for a pediatric burn patient. Our focus was on a 17-month-old female who sustained a 31% total body surface area flame burn to the left side of her body resulting in partial amputation of digits and circumferential burn to her left hand.

**Methods:**

The patient was positioned comfortably with mother present, she was given snacks and toys to engage and distract her. The left hand was positioned palm up flat on padded table, maintained by the patient’s mother and therapist. A 3D scan was then taken to capture the palmar surface of the digits, hand, wrist, and forearm, with each scan taking approximately thirty seconds. The scan was modified using computer-aided design (CAD) software to render a model for 3D printing. The model of the hand and wrist was then printed using a flexible filament thermoplastic polyurethane (TPU). This was then used as a stand-in for the patient’s limb, allowing for precise orthosis fabrication without resistance or opposition from the patient. This method was performed a total of three times as improvements in range of motion were made over a four-week period.

**Results:**

The patient demonstrated a 20-degree increase in radial abduction, a 30-degree increase in palmar abduction, and achieved a neutral wrist position with use of this method. The patient demonstrated an improvement in her Developmental Assessment of Young Children Second Edition (DAYC-2) fine motor subdomain score, indicating an age-equivalency of 17 months up from an initial 10 months.

**Conclusions:**

Compared to traditional hand orthosis fabrication methods, this novel approach allows for better fitting orthoses and is less stressful for the patient. Using a model instead of an active patient enables better contouring and fit of the orthosis, while the flexible TPU material allows for manipulation of the model to optimize joint positioning. It can be used to apply increased pressure on areas with hypertrophic scarring during fabrication, which translates to increased pressure exerted by the orthosis during wear.

**Applicability of Research to Practice:**

This innovative method holds promise as an effective tool for fabricating orthoses for complex hand burns. This method could also be adapted for use in other regions of the body, thereby expanding treatment options for fearful or uncooperative patients.

**Funding for the Study:**

N/A

